# Sustaining, Forming, and Letting Go of Friendships for Young People with Inflammatory Bowel Disease (IBD): A Qualitative Interview-Based Study

**DOI:** 10.1155/2020/7254972

**Published:** 2020-09-03

**Authors:** Alison Rouncefield-Swales, Bernie Carter, Lucy Bray, Lucy Blake, Stephen Allen, Chris Probert, Kay Crook, Pamela Qualter

**Affiliations:** ^1^Faculty of Health, Social Care & Medicine, Edge Hill University, Ormskirk, UK; ^2^Liverpool School of Tropical Medicine, Liverpool, UK; ^3^Institute of Translational Medicine, University of Liverpool, Liverpool, UK; ^4^St. Mark's & Northwick Park, London North West University Healthcare NHS Trust, London, UK; ^5^Manchester Institute of Education, University of Manchester, Manchester, UK

## Abstract

Inflammatory bowel disease (IBD) is an incurable, chronic, gastrointestinal condition that can constrain young people's social relationships. Few studies have specifically explored friendships of people with IBD. This qualitative, participatory study used interviews, photographs, and friendship maps to explore friendships and friendship networks of young people with IBD. An online Young Person's Advisory Group was actively engaged throughout the study. Thirty-one young people participated (*n* = 16 males, *n* = 15 female; *n* = 24 Crohn's disease, *n* = 6 ulcerative colitis, *n* = 1 IBD-unclassified; the mean age at study was 18.7 years; range 14-25 years). Findings present a metatheme “The importance and meaning of friendships” and three interwoven subthemes of “Sustaining friendships,” “Forming new friendships,” and “Letting go of friendships.” Friendship was important to the young people with IBD, providing support, but associated with challenges such as disclosure. Such challenges could be mitigated by clearer conversations with clinicians about friendships and more extensive conversations about friendships and long-term conditions in education settings.

## 1. Introduction

Inflammatory bowel disease (IBD) is an incurable, chronic, relapsing, and debilitating gastrointestinal condition [1], which includes Crohn's disease and ulcerative colitis and IBD-unclassified. It is characterised by uncertainty, unpredictability, and the intrusiveness of symptoms [2, 3]. Research on IBD and young people has been primarily biomedical, with scant attention paid to the psychosocial aspects of living with IBD [4], such that we know little about the challenges of IBD on peer relationships and friendships.

### 1.1. Challenges of IBD

IBD is challenging for young people [5]; common symptoms are diarrhoea, abdominal pain, weight loss, blood in the stools, and fatigue [6, 7]. Treatments for IBD, such as high-dose steroids, can have adverse effects on mood and behaviour. Young people with IBD can experience psychological morbidity [8] and have a reduced Health Related Quality of Life (HRQoL) compared to their peers [9, 10]. Living with IBD increases anxiety and stress among young people and their families, although the reported levels and prevalence of anxiety and depressive symptoms vary across studies [2, 11–16].

The unpredictability of the course of IBD and the embarrassing nature of the symptoms can result in uncomfortable social experiences and constrained social relationships. Young people may conceal their disease [17] from other people in an effort to reduce stigma and people's negative perceptions [18] and avoid rejection by their peers [4]. Having to take time away from their peer group due to symptoms and hospitalisation can heighten loneliness [19, 20] and the feeling of missing out on life [2].

### 1.2. Friendships and Peer Relationships

Friendship is defined as “a relationship between two individuals characterised by support, time, intimacy, trust, affection, and the ability to manage conflict” [21] ^p330^. Young people's friendships are complex, dynamic, and fragile with “deep intrapersonal and interpersonal implications” [22] ^p2^. As they grow up, young people increasingly seek companionship, intimacy, romantic relationships and emotional support, and a sense of identity and belonging from their friends rather than their family [23–25]. Friendships play an increasingly central role in young people's lives as they move into adolescence [26]. Friendships provide a context for the development of values, definition of roles, and refinement of the social skills which are necessary to maintain future relationships [27]. While instability and change are typical during childhood, friendships [28] by adolescence friendships often become more stable with young people seeking intimacy and emotional support from their friends [28, 29].

Friendships and positive peer relationships can provide benefits, supporting young people's social-emotional development [30, 31], mental health [32, 33], self-esteem, and well-being [34] and buffering them from social disconnection and disappointment [35]. However, peer relationships entail costs [36, 37]. While care, concern, trust, loyalty, emotional support, understanding, and intimacy are salient qualities in friendships [38, 39], friendships can also lead to vulnerability, rejection, and unmet expectations [36]. Persistent peer relationship problems can be a source of conflict [36], negatively impacting engagement with education [40] and resulting in higher self-reported levels of depression, anxiety, and loneliness [41, 42]. These issues contribute to poor health well into adulthood [43], with long-term impacts on future friendship quality [44].

Theories of friendship development suggest they are developed and maintained through interconnected factors such as proximity, mutual appeal and positive outcomes [45], shared values, interests, activities, and the level of affection the relationship provides [37]. Changes to those factors can affect their stability [45] as changes can to social networks and health status of members of the dyad [46]. The path of friendship varies; friendships may remain, dissolve, or transform [47].

Few studies have specifically explored friendship quality of people with IBD [48, 49]. Our study is aimed at addressing this deficit by exploring the impact of IBD on the social relationships and psychological functioning of young people (14-25 years) with the condition. We chose to do this by adopting a methodological approach that offered young people with IBD the opportunity to narrate and contextualise their friendship experiences.

## 2. Method

This paper reports qualitative findings from a sequential two-phase study that explored the social lives of young people with IBD. Phase 1 (quantitative) findings are reported elsewhere. The in-depth qualitative study (Phase 2) used an Interpretive Description [50] approach and participatory interview methods.

### 2.1. Aim

The specific aim of the qualitative study was to explore young people's friendships and their friendship networks. The research question underpinning this study was “What experiences of friendship do young people with IBD have and what sort of friendship networks do they have?”

### 2.2. Settings

The settings were clinical areas (outpatient clinics and day case wards) within two university hospitals serving a major city situated in the North West of England; one provides children's services, the other adult services. Using convenience sampling, young people were recruited to participate in the Phase 1 survey via invitation letters sent out from hospital clinics.

### 2.3. Recruitment

Young people aged 14-25 years, diagnosed with IBD, at any point in their disease trajectory beyond the first three months post-diagnosis and who participated in the Phase 1 survey, could express interest in participating in Phase 2. If interested in Phase 2, they were asked to complete an “expression of interest” form which included their preferred means of being contacted (text, phone, or email). We then contacted the young person 2-3 weeks before their scheduled clinic/hospital visit (usually this was 2-3 months following survey completion). If they were still interested in participating, we posted or emailed information sheets that provided more details about the interview and the creative methods (maps, photographs). Young people had the opportunity to discuss whether they wanted to use creative methods, with additional guidance, if needed, being provided. In the week before their appointment, we made arrangements to meet them at their clinic/hospital visit. Consent/assent or a decision to not participate was finalised at the visit and the interview undertaken, if appropriate. Of those who participated in Phase 1, 59 young people provided contact details and expressed an interest in participating in Phase 2. Of these, 28 were unable to participate due to not having a scheduled clinic appointment within the timescale of the study, not attending their clinic appointment or researcher unavailability.

### 2.4. Methods

Our use of multiple qualitative methods (interviews, friendship maps, and photographs) was aimed at promoting conversation and exploration; the young people could choose which method(s) to engage with. All three methods were underpinned by the values of person-centredness and aimed at positioning the young people as experts of their own experience and facilitating their control over what they shared during their interaction with the researcher. Person-centeredness emphasises the importance of building relationships based on mutual respect and understanding [51]. In research practice, the values of person-centredness are expressed through reciprocity, mutuality, and being present in the research relationships [52].

#### 2.4.1. Interviews

The interview was conversational in nature, creating opportunities for the young people to talk about the things that mattered to them; an interview guide was available (see Supplementary File). The interviews, including the dialogue about the maps and photos, were audio-recorded with the young person's permission and later transcribed. All interviews were conducted in a quiet and private setting at a routine clinic or hospital visit (October 2018-April 2019). Researchers had no clinical relationship with the young people, though on some occasions, the interviewer had met the young person in Phase 1.

#### 2.4.2. Friendship Maps

Our approach to friendship maps was based on a participatory mapping technique [53]. Participatory mapping is an interactive approach which uses accessible and straightforward visualisations (e.g., spider diagrams) to enable participants to describe, depict, and theorise how they have represented features on their map through drawing and talking [53]. The visual mapping of friendships enabled the young people to reflect on the evolution of their friendships (e.g., how friendships had started and how long they had lasted) and exploring changing dynamics, the complexities and nuances of friendship practices, and how the young people experienced different forms of friendship (e.g., what made them good friends, what activities they enjoyed doing together, and what difficulties were encountered). The researchers provided the young people who wanted to create a map with the relevant materials (paper, pencils, and coloured pens), and the young people created their map at the start of their interview. Most maps were simple “spider diagrams” (see Supplementary File, Figures 1 and 2) but were creative in using colours as a way of describing friends “I did her in blue because she's often very depressed, unfortunately” (ID29, F25, CD). Young people used line thickness and length to represent the closeness of relationships with less strong lines representing more tenuous friendships such as “I think with this one [line], it's like friends of a friend” (ID17, F15, CD).

#### 2.4.3. Photographs

The use of photographs drew on photo-elicitation technique [54, 55] as a means of enabling young people to use visual images of their friends and friendships to prompt conversation. The use of photographs to trigger discussion enables a repositioning of power [56], and we hoped that this would help the young people feel more in control of what they wanted to talk about. We anticipated that the photographs would evoke emotions, memories, and ideas and unfold different layers of meaning [55]. We asked the young people to take new or use existing photographs as a trigger for our interviews with them. Some young people came prepared with printed photographs and allowed us to retain copies. However, others shared images on phones and tablets; we did not obtain copies of those. Within the interviews, the young people selected which photographs they wanted to talk about and then we used prompts such as “why did you choose this photograph?,” “what is happening?,” and “who is in this photograph?” to elicit their experiences and perceptions. Most photographs were of the young person's friends, taken at memorable gatherings or activities, and enabled them to talk about their lives, in particular to describe different connections and friendships.

### 2.5. Ethical Issues

The study was approved by the North West-Liverpool East Research Ethics Committee (18/NW/0178) and research ethics committees at Edge Hill University and the University of Manchester. Conversations about participation and consent (or assent) were undertaken with the young people (and parents, as appropriate). Specific written consent/assent was gained for the use of photographs and friendship maps.

### 2.6. Young Person's Advisory Group

Key to our commitment to participatory values, an online Advisory Group of ten young people (15-26 years) with IBD contributed to writing the proposal, was influential in determining methods (e.g., suggesting creative methods to use in the interviews) and advising on dissemination. The benefits to the researchers and the study were that research materials and our dissemination were age-relevant. Benefits reported by the young people were that they could see the impact that their suggestions had made to the progress and quality of the study.

### 2.7. Analysis

Interview data were subjected to an iterative, interpretive approach in line with Interpretive Description [50], with analysis being supported through the use of field notes and synopses of each young person's data. Analysis initially focused on each young person's data (interview, friendship map or photographs). Each interviewer (AR-S, BC, LBr, and LBl) who had personally undertaken the interview read and reread the transcription to refamiliarise themselves with the data and created a synopsis for each young person. Each interviewer identified preliminary themes and meanings and proposed tentative theoretical connections to reflect the social relationships and psychological functioning of young people. Then, working collaboratively, we shared these themes and ideas with each other and started to group the data in different ways (e.g., age, gender, and disease severity) looking for new patterns, resonances, and differences and a deeper understanding. Some ideas were dropped, but others were strengthened. The highly reflexive process we followed is aimed at enhancing the rigour of the study by ensuring that we challenged any preconceptions, looked deeper for stronger interpretations, and were able to fully justify any claims made [57]. Part of this process involved two researchers (AR-S and BC) working across all interviews to ensure an overall perspective was achieved. This iterative, collaborative process allowed us to reach consensus and create the metatheme “The importance and meaning of friendships” and three interwoven subthemes of “Sustaining friendships,” “Forming new friendships,” and “Letting go of friendships.”

## 3. Findings

Thirty-one young people participated; of these, five created friendship maps and six utilised photographs within their interviews. Interviews ranged in length from approximately 20 to 60 minutes. In seven of the interviews, at the request of the young person, a parent was present, often informally contributing by clarifying the researcher's questions, supplementing the young person's response, or reminding the young person of activities or events.

Sixteen participants were male, and 15 were female; on average, the age at study was 18.7 years (range 14-25 years); the mean age at diagnosis was 14.4 years (range 8-23 years). Most were White. Most (*n* = 21) had been diagnosed five years or fewer. Twenty-four young people had Crohn's disease (two had stomas), and seven had colitis (six with ulcerative colitis and one with IBD-unclassified). Seventeen were classified as being in remission (a decrease or disappearance of the symptoms), nine had mild disease severity, and five had moderate disease severity (Table 1).

The findings presented in this paper focus on the metatheme “The importance and meaning of friendships” and three interwoven subthemes of “Sustaining friendships,” “Forming new friendships,” and “Letting go of friendships.” Another paper (Authors, in press) explores young people's decisions about disclosing their diagnosis to friends.

Anonymous quotations are linked to interview number (e.g., ID2), gender, age (F12), and conditions: CD (Crohn's disease), UC (ulcerative colitis), or UnC (IBD-unclassified).

### 3.1. The Importance of and Meaning of Friendship to Young People with IBD

Friends and friendship groups that provided companionship, closeness, connection, acceptance, and fun were important to the young people in this study. Friends provided the young people with opportunities to escape and put their IBD into the background, particularly when they encountered tough times associated with flare-ups, hospitalisation, treatments, surgery, and the ongoing symptoms of IBD. One young person explained:

My friends are great… if you've been off a couple of weeks or even if you've got a tube sticking out of your nose…. it's just, ‘oh what's that?' and I explain quite quick and they're like ‘oh, sure, fine ‘.. back to normal, so that's great (ID3, M15, CD).

Having “people that I can have fun with” (ID17, F15, CD) was important. Equally important was that those friends were “honest with each other” (ID19, M22, CD). Having friends who would be there for them, “to talk to like if I'm not well or something” (ID26, M16, UC) and whom they could trust was essential:

I can talk to them and I trust them a lot, I trust them all, I can tell them anything and they tell me anything (ID17, F15, CD)

Friends who understood and accepted the disruptions associated with IBD and did not blame the young person for those disruptions were important:

My best friend …. Like, I'll be at her house and I'll be like ‘oh my tummy hurts. I'm going to the toilet'. She's like, ‘don't worry I know you're going to be an hour. I'll be waiting' (ID28, F19, CD).

Some young people mentioned stigma and rejection as issues, reflecting that some people mistakenly believed that IBD was a transmittable disease, noting that “the word disease scares them” (ID6, F16, CD):

… some people don't like it or just because you are different they don't want to see you or speak to you because you've got a disease. (ID1, F16, CD)

Acceptance and “having friends who are very open” (ID11, F21, UC) were core to meaningful friendships and the “sort of people” (ID11, F21, UC) that were valued within the young people's lives. Such friends “grew” with the young person and came to “understand” and accept the stranger aspects of IBD, such as tube-feeding, and could act “normal” even if they had little experience of such things and shared responsibility for sitting with their friend “they would swap each day” (ID27, F15, CD).

Although IBD did not necessarily dominate their lives, young people felt that their IBD was always present, “it's [Crohn's] always there. I think that's the main thing that needs to be said. It's never gone” (ID12, F23, CD).

### 3.2. Sustaining Friendships

Sustaining existing friendships was important. Having friends meant a lot to the young people; close and trusted friends were key to feelings of connection. Some talked about how some friends who had known them before they got sick “kind of went through that kind of journey with me” (ID22, M24, CD).

Good friendships were reciprocal. Young people acknowledged that when their friends faced difficulties, they were “there” for them: “[I] allowed her to complain like a lot, I tried to be supportive and then the next day I will dump all my load on her and she will help” (ID29, F25, CD). Many friendships were strengthened as a result of IBD with young people describing their friends becoming “protective” and closer. This closeness did not necessarily require friends to fully understand what the young person was going through:

… they are understanding although they don't understand… they appreciate that I have difficulties sometimes with food or change of bags or whatever… (ID25, M21, CD).

However, some young people found it difficult to sustain friendships; one explained she had “gone into a shell, making it difficult to maintain friendships” (ID21, F20, CD) whereas others found that “It's a lot harder to keep in contact with people” (ID29, F25, CD) or found “it difficult to make plans because… I hate letting them down (ID10, M23, UC). This extra effort to sustain friendships was reflected in the stories they shared:

I was really thankful to them for still being friends with me, I'd been off for quite a while, whilst it didn't become stronger, I knew I could rely on them to remain friends whatever, I'd do the same for them obviously, because that's what friends do (ID3, M15, CD).

IBD created physical and emotional limitations that impacted on young people's ability to maintain friendships. The disruption to the taken-for-granted social aspect of eating was a limitation that many of the young people talked about. This disruption ranged from the apparently modest disruption of sitting in the cinema and “just smelling the popcorn” (ID1, F16, CD) to the challenge of trying to fit special diets in around family routine and time with friends, for example “they say ‘Oh let's go get something to eat' and I can't, I've got to have a shake in half an hour” (ID7, M14, CD).

Being able to join in activities they enjoyed with their friends meant working around IBD-related restrictions. Sometimes, that involved self-limiting behaviour to ensure they fit in, such as at university, where going out and drinking were part of social activities:

I won't drink as much as they do… I want to be in a good state in case my bag does come off, which has happened because I dance quite mad…to change it if I need to (ID25, M21, CD).

Some young people talked of being onlookers “just watching them all like having fun” (ID6, F16, CD) with fatigue limiting their ability to participate in normal social activities:

I used to get extremely fatigued so a day at school 9-3:30pm would just exhaust me. So then maybe to think that everyone would go out and do an activity, I'm too tired, I can't go and join in on that. (ID9, F21, UC)

Many went out less often than their friendship group “… If I go out like four or five times a week, week after week, it does put toll on me. So I see them once a week” (ID30, M24, CD). Instead, they often sustained connections with their friends through social media or by phone as “just a phone call. It just completely changes your whole, the whole, feeling your whole surroundings” (ID30, M24, CD).

Good friends were described as those who understood and accommodated young peoples' particular needs, acknowledged their fatigue, and chose activities they could engage in. Tricky conversations with friends, such as those about types of social activities or toilets, often resulted in good friends being accommodating in their plans, “we'll go to this place' cause the toilet's actually quite nice there, you can use it” (ID22, M24, CD). Despite friends being accommodating, some young people felt anxious about worst-case scenarios and “what if” situations when they were out with friends, “It's the sort of fear of what if suddenly I don't feel very well – what if I suddenly have to run to the toilet…” (ID24, M20, CD).

### 3.3. Forming New Friendships

Forming new friendships was important to the young people. Some talked of feeling confident about making new friends and did not think that their IBD made much difference, and it was a case of “finding the time” (ID11, F21, UC). However, others expressed concerns about how their IBD may influence the perception of them by potential friends. While many talked of the importance of preserving a sense of being “normal” in relationships that were developing, they also expressed a desire to establish friendships with those people who would be understanding about the difficulties that may accompany their IBD:

I think I'll naturally be more drawn to be closer with people that take it seriously…. Obviously, I wouldn't want to stay friends with someone who just forgets the differences between me and them… in general….my illness isn't really at the forefront of my mind when making friends (ID20, M24, UC).

Situations and transitions such as moving schools, starting college or university, starting work, or becoming involved with new social or group activities were not only opportunities to form new friendships, but also situations where IBD might constrain their chances of making new friends. Worries often reflected the degree to which the young people's identity was linked to their IBD. Many young people wanted to project an identity in which they would be seen as being the “same as” peers:

Like everyone I want to be perceived as normal, not average but you know the same as everyone else. I don't want it to be that I come with a list of exceptions (ID9, F21, UC).

Projecting “normal” meant that some young people kept their diagnosis to themselves because of fears that it would change their interactions with friends, noting that “I don't think [talking about] bowel movements is the way to go” (ID29, F25, CD) and explaining “I don't want people to start feeling sorry for me or change the way they treat me because of it” (ID21, F20, CD). The reactions of people where a friendship was in an early stage of development were a real concern, especially in relation to the potential reaction to knowledge about their disease:

The few times I have brought up my colostomy and Crohn's in Uni… it just turns people feeling sorry for me and which then puts me off talking about it more because I don't want them to feel sorry for me (ID25, M21, CD).

This led to concerns that their identity would be framed by their condition and that potential friendships may stem from people “feel[ing] sorry for me – a sympathy friendship” rather than a true friendship. The young people wanted to make sure they were “not being defined by it [their condition]” (ID10, M23, UC) and wanted friends who knew they were “more than Crohn's” (ID21, F20, CD).

### 3.4. Letting Go of Friendships

Although IBD had not resulted in the loss of friendships for some young people, “I don't think I've ever lost a friend because of the effects of Crohn's disease” (ID24, M20, CD), others recalled friendships broken by their IBD or times when they felt let down by friends who had failed to understand the magnitude, variety, and impact of symptoms. Some young people talked of how people they had considered to be friends “couldn't be bothered” with them anymore. This often led to questioning the value of those particular friendships:

I've lost a few friends… because they haven't understood or they'll try and push the matter… They don't realise it's quite hard at times. (ID30, M24, CD).

Some young people talked of how a response to their IBD could negatively affect a friendship:

I remember her being like….cringey I don't want that [IBD]… you just know who your friends are when you get diagnosed and stuff like that happens (ID21, F20, CD).

Friends “making fun” or making negative or insensitive comments regarding their IBD were difficult to deal with, as was a perception that the young people were somehow using their illness as an excuse to gain benefits such as “sympathy” or the opportunity to “miss exams,” even when these supposed benefits “made no sense when you think about it” (ID12, F23, CD).

Negativity from people they had hoped to rely on for support and comfort, feeling abandoned, and rejected by friends were distressing for young people who were already dealing with the physical and emotional consequences of IBD. Some talked of this as a tipping point, a point at which managing their IBD became really difficult. However, the passage of time allowed most of the young people to see the loss of friends in context despite the pain and distress they experienced at the time:

… I kind of feel like I'm better off and like I know who is there for me and who's not. So I'm not really bothered. At the time, you know, it was the worst thing in the world (ID28, F19, CD).

## 4. Discussion

This qualitative, participatory study facilitated young people with IBD to share their experiences of friendship. They recounted the importance of friendships and the personal and emotional challenges of sustaining, forming, and letting go of friendships, adding depth to earlier reports of how IBD may impact on a young person's friendships and social relationships [48, 49]. Although all young people face challenges associated with friendships, the awareness of the importance of friendships was heightened for these young people with IBD by the stress, unpredictability, uncertainty, and stigma associated with the condition, its trajectory, and treatment. Our findings add depth and insight about the nuances of adolescent and young adult friendships which is missing in a previous work [4, 13, 18].

Young people with IBD in the current study talked of the importance of having good friends, friends who were understanding and accepting, friends they could trust and talk to and who made them laugh, and friends who helped them feel socially connected even during illness flares. Those qualities are ones that any young person may view as positive characteristics of a friend [58]. But as with other studies of young people with IBD or other long-term conditions, such qualities were particularly important [59, 60], unsurprisingly considering the challenges they reported about life with IBD.

Interconnection is necessary to develop and maintain friendships [45], and this is often established through being together and shared activities. However, for the young people in the study, establishing and sustaining interconnection were sometimes difficult and, as with other studies, opportunities to make new friends were sometimes diminished [6]. Absence from school, college, or work or symptoms restricting social activities meant that some young people with IBD experienced a sense of isolation. This is at a time when friendships are particularly important, as young people are learning how to navigate, create, and sustain peer group relationships [61]. Other studies addressing the impact of long-term conditions reveal the importance of friends in mitigating the social difficulties, restrictions of the condition, and sense of isolation [62, 63]. We found that while maintaining a sense of being “normal” and keeping pace with peers were important for many young people, they developed tactics and adaptions to work around the physical and emotional limitations imposed by their IBD, as found in other studies of young people with a long-term health condition [62, 64, 65].

Whether through choice or not, the young people tended to concentrate on relationships with their closest, most supportive, and understanding friends who could accommodate the strain and difficulties associated with their IBD. The breakdown of a friendship might be potentially problematic for young people with IBD who may be perceived as, and indeed may perceive themselves as, different and not conforming with social norms or expectations, as seen with other studies of long-term conditions [66]. As was found in previous studies, young people acknowledged the fragility of friendships and the costs of rejection and unmet expectations [36] but acknowledged the benefits of robust relationships. The loss of some friendships resulted in some young people reporting smaller friendship networks; other studies have also reported ill health impacting on friendships and resulting in smaller friendship networks [13]. Falci and McNeely [67] note that smaller networks can be associated with low perceptions of friend support and belonging but this did not seem to be the case in our study, as the smaller networks were often cohesive and supportive. Our findings align with the research on social networks which emphasise the dynamic landscape of friendships [68]; our young people acknowledged this fluidity in friendships but found conflict perceived as being connected to their IBD difficult to contend with.

Social support after being diagnosed with a long-term condition is important [66], especially for those with stigmatised identity [69]. Some young people in the current study concealed their IBD from friends, while others downplayed the seriousness of their condition. Limiting disclosure and explanations about the “gory detail” are aimed at both protecting their friends and minimising the risk of rejection [17], although a consequence of this could be limiting access to the support that close friends can offer. Friends with a better understanding of IBD and its implications could help sustain social support and potentially mitigate some of the impact of living with IBD. As seen with adults with IBD, young people who were more open about their IBD to a close and trusted network of friends often experienced positive responses: friends developed an understanding of the condition and accommodated restrictions [69] and often became conduits to broader peer relationships [66]. This connects to the work of Flynn et al. [44] who found young people with a supportive network are better able to form reliable and compassionate friendships. Young people in our study described certain friendships as being closer and more meaningful since disclosure [17], possibly reflecting a type of posttraumatic growth, sometimes experienced by young people after trauma exposure [70].

Recommendations arising from our study include the need for better support for young people in terms of managing their concerns and experiences of sustaining, forming, and letting go of friendships. Opportunities exist for conversations to occur that ensure that young people, their friends, and wider peer group can explore friendship, IBD and other long-term conditions. As a result of our findings and in collaboration with our online Advisory Group, we developed a resource to support such conversations (see “Telling My Friends” https://ehu.ac.uk/CrohnsorColitis).

### 4.1. Limitations

Our findings need to be considered in the context of some limitations. The recruitment of the sample for this phase of the study was from two hospitals within the same city; most participants identified as White, affecting the representativeness of our findings for a broader population. Although only about 20% of the young people created maps and/or shared photographs, we do not perceive this as a limitation. Giving young people choice over which aspects to participate in was fundamental to our person-centred approach, and it generated a supportive context for the interviews. Although parental presence may have shaped what was shared by some young people [71], parental presence was always at the request of the young people, and it always appeared supportive and nondirective. Fewer young people with colitis were interviewed than those with Crohn's, and no young people with severe disease activity chose to participate. Further research exploring the experiences of this population is needed.

## Figures and Tables

**Table 1:** Key demographics of participants.

ID	Gender	Age years (age diagnosed)	Diagnosis	Surgery (stoma)	Disease activity^(1–4)^
2	Female	14 (11)	Crohn's	No	Mild^4^
4	Male	14 (13)	Crohn's	No	Remission^4^
7	Male	14 (12)	Crohn's	No	Mild^4^
3	Male	15 (9)	Crohn's	Yes (stoma)	Remission^4^
5	Male	15 (9)	Crohn's	Yes	Mild^4^
16	Male	15 (11)	Crohn's	No	Remission^4^
17	Female	15 (12)	Crohn's	No	Moderate^4^
27	Female	15 (11)	Crohn's	No	Remission^4^
1	Female	16 (11)	Crohn's	No	Mild^4^
6	Female	16 (13)	Crohn's	No	Remission^4^
14	Female	16 (10)	Crohn's	Yes	Remission^4^
15	Male	16 (15)	Crohn's	No	Remission^4^
28	Female	19 (8)	Crohn's	Yes	Remission^3^
21	Female	20 (19)	Crohn's	No	Remission^3^
24	Male	20 (12)	Crohn's	No	Mild^3^
31	Female	20 (12)	Crohn's	Yes	Moderate^3^
23	Male	21 (14)	Crohn's	No	Remission^3^
25	Male	21 (20)	Crohn's	Yes (stoma)	Remission^3^
19	Male	22 (21)	Crohn's	Yes	Remission^3^
12	Female	23 (15)	Crohn's	Yes	Mild^3^
22	Male	24 (16)	Crohn's	No	Moderate^3^
30	Male	24 (19)	Crohn's	No	Remission^3^
18	Female	25 (23)	Crohn's	Yes	Mild^3^
29	Female	25 (23)	Crohn's	Yes	Mild^3^
13	Female	16 (15)	Ulcerative colitis	No	Remission^1^
26	Male	16 (12)	Ulcerative colitis	No	Mild^1^
9	Female	21 (8)	Ulcerative colitis	No	Remission^2^
11	Female	21 (16)	Ulcerative colitis	No	Moderate^2^
10	Male	23 (20)	Ulcerative colitis	No	Moderate^2^
20	Male	24 (22)	Ulcerative colitis	No	Remission^2^
8	Male	14 (12)	IBD unclassified	No	Remission^1^

^1^Paediatric Ulcerative Colitis Activity Index (PUCAI); ^2^Simple Clinical Colitis Activity Index (SSCAI); ^3^Harvey-Bradshaw Index (HB Index); ^4^weighted Paediatric Crohn's Disease Activity Index (wPCDAI).

## Data Availability

Data are not freely available.
