# Supplementary material for: Sustaining, Forming, and Letting Go of Friendships for Young People with Inflammatory Bowel Disease (IBD): A Qualitative Interview-Based Study
Source: Int J Chronic Dis. 2020 Sep 3;2020:7254972. doi: 10.1155/2020/7254972 (PMC7487095; doi:10.1155/2020/7254972)
Supplement: Supplementary Materials — The interview guide and friendship maps 1 and 2 are provided as a supplementary file. [file 7254972.f1.docx]

Note these statements are prompts which will need framing to make more personal/relevant to individual participants and will follow on from preliminary talk re study, assent/consent etc.

1. Tell me a bit about yourself

- Home/family
- School, college, university, work etc
- What do you enjoy doing?

1. If you were writing down a list of friends, who would be on that list

- Please, tell me something about those friends
- What sort of things do you usually do with these friends?
- What do you enjoy most about being with these friends?

1. If you were taking a picture or pictures or imagining a picture(s) that summed up you and your friends what would that picture(s) be of?
2. Do you have a friend to whom you feel closest? Or are all of your friendships equally close?

- For those who have a best friend: How long have you been friends with one another? Any ‘romantic’ relationships
- What do you value most about this friendship?

1. Do you have different groups of friends?

- How long have you known them?
- How did you meet them?
- Have your friendships changed over the past few years/since school/college? In what ways? Why?

1. What is the thing you value most in a friendship?

- Is it spending time together, laughing at the same kind of things, being able to tell them things? Or all those things?

1. Do you find any aspect of your friendships difficult or challenging?

- Like telling your friends about how you feel? Or finding time to spend with them?
- Do you work hard at your friendships?

1. Do you think having IBD has had an impact on your friendships (… including actual/potential romantic relationships)?

- Do you think it has an impact on the way that you make friends? Or keeping friendships going?
- Did anything in your friendships change when you found out you had IBD?
  - If yes, what kind of things? In what ways did the friendship change? How did you feel about this?
- Do things change with your friends when/if your IBD gets worse?
  - If yes, in what kind of ways?
- Are there any activities your friends enjoy which you cannot take part in? How do you feel about this?
- Do you think that your IBD might/does get in the way of romantic relationships? How?

1. Would you like to change anything about your current friendships?

- If yes, what kind of things?

1. Do you see anything in the future that makes you worried about your friendships?

- If yes, what kind of things (e.g. leaving home)?

1. Anything else about your friendships that you want to tell us about? Anything that we didn’t ask?

Closing interview

- Thanks for taking part in the interview
- Can we keep the photos/map (if applicable)
- Are you OK? Has anything from the interview been upsetting? Here’s the Thank You and Helpful Information Sheet (plus other exit talk as per protocol).
